# Intra-Examiner Reliability and Validity of Sagittal Cervical Spine Mensuration Methods Using Deep Convolutional Neural Networks

**DOI:** 10.3390/jcm13092573

**Published:** 2024-04-27

**Authors:** Mohammad Mehdi Hosseini, Mohammad H. Mahoor, Jason W. Haas, Joseph R. Ferrantelli, Anne-Lise Dupuis, Jason O. Jaeger, Deed E. Harrison

**Affiliations:** 1 Ritchie School of Engineering and Computer Science, University of Denver, Denver, CO 80208, USA; mohammadmehdi.hosseini@du.edu (M.M.H.); mmahoor@du.edu (M.H.M.); 2 Dreamface Technologies LLC, Centennial, CO 80111, USA; 3 CBP Non-Profit, Inc., Eagle, ID 83616, USA; drjasonhaas@gmail.com (J.W.H.); joe.ferrantelli@postureco.com (J.R.F.); 4 PostureCo, Inc., Trinity, FL 34655, USA; annelise.dupuis@postureco.com; 5 Community Based Internship Program, Associate Faculty, Southern California University of Health Sciences, Whittier, CA 90604, USA; drjaeger@spineandposture.com

**Keywords:** cervical lordosis, reliability, computer vision, deep convoluted neural networks, sagittal balance, predictive models

## Abstract

**Background:** The biomechanical analysis of spine and postural misalignments is important for surgical and non-surgical treatment of spinal pain. We investigated the examiner reliability of sagittal cervical alignment variables compared to the reliability and concurrent validity of computer vision algorithms used in the PostureRay^®^ software 2024. **Methods:** A retrospective database of 254 lateral cervical radiographs of patients between the ages of 11 and 86 is studied. The radiographs include clearly visualized C1–C7 vertebrae that were evaluated by a human using the software. To evaluate examiner reliability and the concurrent validity of the trained CNN performance, two blinded trials of radiographic digitization were performed by an extensively trained expert user (US) clinician with a two-week interval between trials. Then, the same clinician used the trained CNN twice to reproduce the same measures within a 2-week interval on the same 254 radiographs. Measured variables included segmental angles as relative rotation angles (RRA) C1–C7, Cobb angles C2–C7, relative segmental translations (RT) C1–C7, anterior translation C2–C7, and absolute rotation angle (ARA) C2–C7. Data were remotely extracted from the examiner’s PostureRay^®^ system for data collection and sorted based on gender and stratification of degenerative changes. Reliability was assessed via intra-class correlations (ICC), root mean squared error (RMSE), and R^2^ values. **Results:** In comparing repeated measures of the CNN network to itself, perfect reliability was found for the ICC (1.0), RMSE (0), and R^2^ (1). The reliability of the trained expert US was in the excellent range for all variables, where 12/18 variables had ICCs ≥ 0.9 and 6/18 variables were 0.84 ≤ ICCs ≤ 0.89. Similarly, for the expert US, all R^2^ values were in the excellent range (R^2^ ≥ 0.7), and all RMSEs were small, being 0.42 ≤ RMSEs ≤ 3.27. Construct validity between the expert US and the CNN network was found to be in the excellent range with 18/18 ICCs in the excellent range (ICCs ≥ 0.8), 16/18 R^2^ values in the strong to excellent range (R^2^ ≥ 0.7), and 2/18 in the good to moderate range (R^2^ RT C6/C7 = 0.57 and R^2^ Cobb C6/C7 = 0.64. The RMSEs for expert US vs. the CNN network were small, being 0.37 ≤ RMSEs ≤ 2.89. **Conclusions:** A comparison of repeated measures within the computer vision CNN network and expert human found exceptional reliability and excellent construct validity when comparing the computer vision to the human observer.

## 1. Introduction

The burden of spine injuries and chronic spine pain for patients and society is tremendous and a growing global concern. Diagnosis, treatment, and long-term consequences of spine conditions are the single greatest cause of disability due to musculoskeletal disorders globally [1]. Diagnosis and intervention for spine conditions vary greatly due to socio-economic conditions, access to current treatment methods and techniques, presence of prior and concomitant conditions, and past interventions, whether conservative, therapeutic, or surgical [1,2,3]. Interventions for spine conditions each have varying potential benefits and costs for the patient and society [4,5,6]. Finding efficacious, validated, repeatable, and reliable diagnostic and therapeutic or surgical methods to resolve and improve spine pain is of great benefit to the individual as well as global populations [7,8,9,10].

Technological advances in spine pain diagnosis and treatment are necessary to reduce cost, improve outcomes, increase efficiency for the facilities and clinicians as well as reduce poor outcomes that may require additional care [11,12,13]. Technology can make the process of spine condition diagnosis more efficacious [14,15,16]. For example, previously, we presented a machine learning deep convoluted neural network (DCNN or CNN) that demonstrated that computer vision (CV) is superior to human measurement of spine displacements [17]. CNNs or DCNNs evolved from traditional artificial neural networks, having their origins based on the understanding of the visual cortex of animals, and are commonly used to identify imaging and video patterns. In this investigation [17], following thousands of evaluations, the program model and software found perfect reliability via intra-class correlation (ICC) and linear regression R^2^ values. Further, the root mean squared error (RMSE), which measures the average difference between a statistical model’s predicted values and the actual values, was zero. To our knowledge, no prior program, study, or software has demonstrated this perfect accuracy and repeatability for spine mensuration. This technology could prove critical for improving the biomechanical analysis of normal and abnormal spinal configurations and could significantly alter treatment for many treating physicians.

This current investigation is a continuation of a previous investigation that we performed, where we provided comparisons with the current CNN model to other CNN models [17]. Herein, we present unique findings from intra-examiner measurement reliability with a repeated measures design using a highly skilled examiner (human) on an original retrospective database of lateral cervical radiographs. Secondly, we present the machine learning CNN system using the same repeated measures design on the same X-ray images to simultaneously investigate reliability with concurrent construct validity against the human study to determine abnormal cervical sagittal spine configuration using intersegmental, regional, and global analyses. We hypothesize that while both the human and CNN systems will have excellent intra-examiner reliability, the CNN will be perfect to near perfect and the construct validity will be high.

## 2. Materials and Methods

### 2.1. Radiographic Image Selection Inclusion Criteria

This study retrospectively obtained 254 consecutive lateral cervical radiographs from a clinical chiropractic practice that required radiographic examination of presenting patients between 1 January 2021 and 10 September 2021. Due to the retrospective nature of our collected material, our design is exempt from IRB approval under section 45 CFR 46.101(b)(4). See https://www.hhs.gov/ohrp/regulations-and-policy/decision-charts-pre-2018/index.html#c5 (accessed on 15 April 2024). The patient ages at the time images were obtained ranged from 11 years old to 86 years old, with 108 males and 146 females. To mimic “real-world clinical practice” and better test reliability and validity of a human and the CNN system, we included all radiographs only if the C1–C7 region was visible. The radiographic images were obtained retrospectively using the PostureRay^®^ radiographic documentation system (PostureCo, Inc., Trinity, FL, USA). This patented software system is commonly used by clinicians to streamline radiographic spinal alignment documentation workflows.

### 2.2. Radiographic Image Selection Exclusion Criteria

Radiographic image exclusion criteria were based on two criteria: (1) if C1–C7 were not clearly visible to the eye or were cut off on the image, and (2) if surgical devices or other obvious artifacts were visible on the X-ray image. No other exclusion criteria were used, and all types of spine degenerative changes and altered sagittal alignment were allowed. As a result, 254 radiographic images were included in this study sample. These retrospective X-ray images were not part of the original dataset used to train the neural networks, representing the first exposure of the trained network to these data. The original Deep CNN was trained and evaluated on 24,419 annotated unique patients’ lateral cervical X-rays, digitized by an expert clinician. It is notable that 95 percent of these data were used for training and 5 percent for validation. For more details, see [17].

### 2.3. Intra-Examiner Reliability and Construct Validity Design

To evaluate intra-examiner reliability and construct validity of the trained network’s performance, any prior digitization annotation markings and measurements were cleaned from the images prior to clinician processing. Two blind trials of digitization were then performed by a trained clinician (JRF) with a two-week interval between trials. The anatomical digitization points used were as follows:

1. Three points on C1: anterior tubercle, midpoint C1 at the posterior margin of the dens, and midpoint of the posterior spinal laminar line.
2. C2–C7 digitization consisted of four points per vertebra: anterior superior, posterior superior, anterior inferior, and posterior inferior vertebral body margins.

In this current study, lateral cervical measurements obtained in the PostureRay^®^ software 2024 were derived from the following anatomical digitization points:

1. Atlas plane relative to horizontal;
2. Segmental posterior body tangent relative rotational angles (RRAs);
3. Cobb analysis using vertebral endplate angles;
4. Segmental relative linear translation distances;
5. Global posterior tangent absolute rotational angle from C2 to C7;
6. Global sagittal horizontal translation alignment of C2 relative to C7.

**Figure 1:**
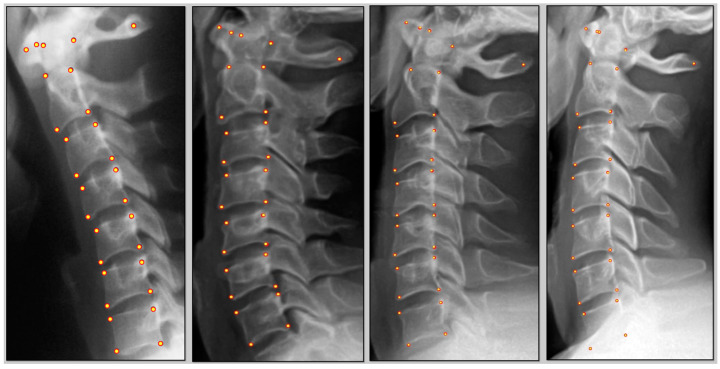
Four examples of model prediction (yellow points) versus human annotation (red points). The accuracy of the model in predicting the landmark points is comparable with the human annotator.

After each trial, the data were remotely extracted from the examiner’s PostureRay^®^ 2024 system for data collection and sorted based on gender and stratification of degenerative changes. The clinician responsible for digitization did not have access to the raw data, nor were they involved in interpretation of statistical analysis at any point. Figure 1 shows several images with the landmark points used in this investigation.

### 2.4. Measurement Variables Reported

In the following section, we describe our methodology in more detail and the parameters and measures used for evaluating the model’s reliability. We originally trained a CNN-based deep neural network model using more than 24 K sagittal cervical spine X-ray images and the provided anatomical landmark points. The landmark points were labeled by expert humans and utilized to train a robust model. The images included three types of poses: poses including normal neutral lateral cervical, lateral cervical extension, and lateral cervical flexion. The details of our model design and implementation can be found elsewhere [17]. In the current study, we randomly selected 254 consecutive images meeting the above inclusion criteria, not included in the training set, to automatically predict the landmarks. In the next step, an expert human corrected the location of the falsely predicted landmark points. It is noteworthy that we repeated this experiment at two different times (two weeks apart) to be able to analyze the intra-examiner agreement during the first and second rounds of landmark corrections.

To evaluate the agreement between the model and the expert human, and the expert’s agreement in the first and second experiments, 18 translational and rotational measurement variables are extracted. The variables are as follows:

- ARA (°): Absolute rotational angle refers to the overall curve of the cervical lordosis. It is computed as the angle between the vertebrae C2 and C7. It is the angle between two straight lines, where they intersect each other. The first line passes through the posterior inferior and posterior superior body corners of vertebra C2, and the second line is the line that intersects the posterior inferior and posterior superior vertebral body corners of C7.
- RRA (°): Relative rotational angle is the angle between two consecutive vertebrae. To calculate this angle, we draw the lines passing through the posterior superior and posterior inferior of any vertebral body corners and then calculate the angle where they cross each other. Thus, creating the slope or the first derivative of the curve when expanded across the vertebral column.
- KA (°): This represents endplate cross-sectional angle, where for two adjacent vertebrae, we draw the lines that pass the anterior inferior and posterior inferior body corner of each vertebra body as well as the anterior superior and posterior superior body corner and then calculate the angle of their intersection. This measurement is considered less reliable due to the nature of degenerative change at the endplate, which can make two like points difficult to assess.
- ST (mm): Denotes segmental translations. Like RR and KA features, it is calculated for any pair of adjacent vertebrae and determines the forward or backward translation along the z-axis between two neighboring vertebrae. Positive value means anterior translation, and a negative value means posterior translation relative to the adjacent segment.
- C1H (°): Demonstrates the atlas plane angle relative to true horizontal and is measured as an angle between a horizontal line and vertebra C1.
- TR (mm): The translational distance of the C2 posterior superior body corner relative to a vertical line drawn superiorly from the C7 posterior inferior body corner is considered as the translation measure in millimeters.

It is notable that the variables RRA (a.k.a. RR), KA, and ST are calculated for any two consecutive pairs of cervical vertebrae from C2 to C7 and, thus, provide a segmental stability analysis for both rotations and translations.

### 2.5. Statistical Analysis

Using the Python (3.8.10) libraries, including NumPy (1.23.4), Pandas (1.5.3), Scikit-learn (1.2.1), SciPy (1.10.0), and Pingouin (0.5.3), statistical analysis of human intra-examiner and CNN reliability was performed on both trials to assess reliability data as well as to compare the CNN measurements vs. the clinicians. Additionally, real-world construct validity was evaluated by the clinician after the network automatically predicted digitization localizations. In this process, the clinician adjusted the anatomical predictions when necessary, ensuring correct anatomical locations. This allowed tracking of rotations and translations of the computer-predicted digitized locations compared to the ground truths determined by the clinician. PostureRay^®^ calculated rotations and translations of clinical lines of mensuration based on these digitization points, and statistical analysis was performed on these measurements. 

As a detailed reliability assessment of the analytical measures, in addition to the mean error and standard deviation of errors of measurement, we report the root mean squared error (RMSE), intraclass correlations (ICC), and linear regression R^2^ measures in this study. Note: (1) the RMSE measures the average difference between a statistical model’s predicted values and the actual values. (2) The intraclass correlation (ICC) is a descriptive statistic of reliability between 2 or more datasets where quantitative measurements are made on units that are organized into their respective groups. The ICC ranges from 0 to 1 and describes how strongly units in the same group compare to one another, where 1 is perfect. (3) Finally, the R^2^ linear regression analysis was used to compare the two measured variable sets for human vs. human measures, CNN vs. CNN measures, and human vs. CNN measures in order to determine the statistical fit and percentage variation between the two measurements for within and between each of the methods. In general, interpreting the relative strength of a relationship based on its R^2^ value is the following: (1) none or very weak effect size R^2^ < 0.3; (2) a weak effect size 0.3 < R^2^ < 0.5; (3) a moderate effect size is 0.5 < R^2^ < 0.7; (4) a strong effect size is given by R^2^ > 0.7; and (5) R^2^ = 1.0 is perfect agreement [18].

## 3. Results

**Figure 2:**
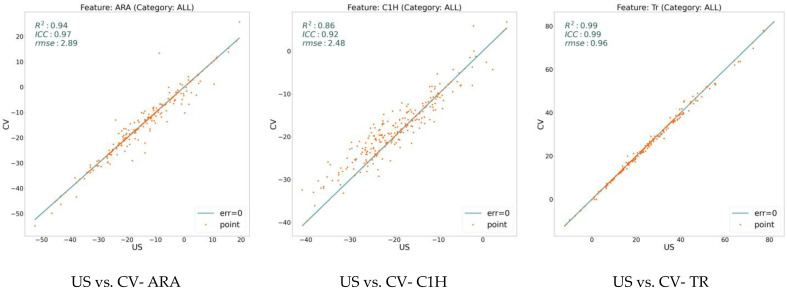
Error analysis between human expert (US) and the model (CV) over three general features, including ARA, C1H, and TR. While x-axis shows the feature value calculated based on the expert’s annotation, y-axis determines the value based on the model’s prediction. The points on the line have zero errors.

**Figure 3:**
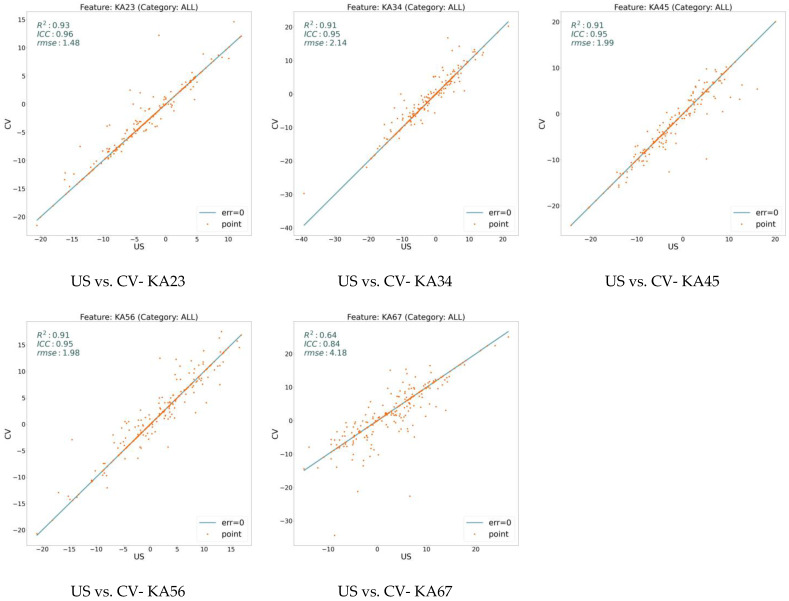
Error analysis between human expert (US) and the model (CV) over KA feature. While x-axis shows the feature value calculated based on the expert’s annotation, y-axis determines the value based on the model’s prediction. The points on the line have zero errors.

**Figure 4:**
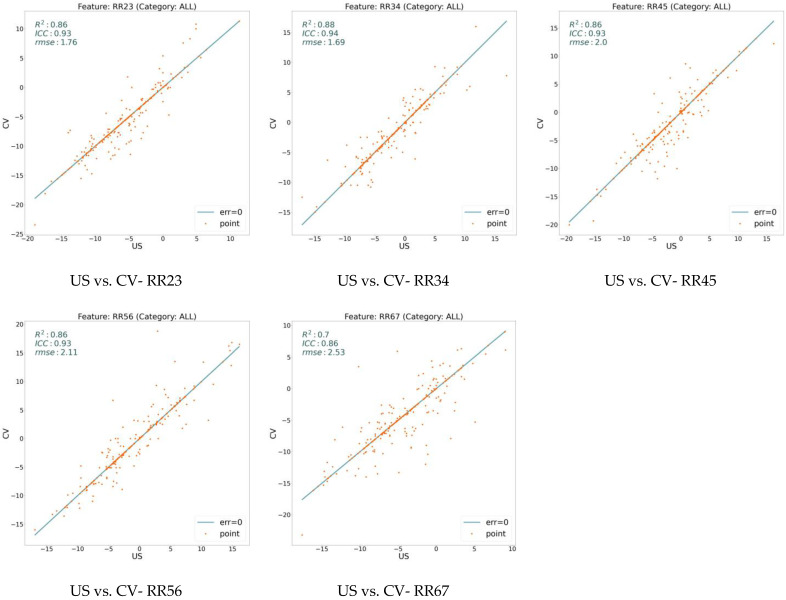
Error analysis between human expert (US) and the model (CV) over RRA (RR) feature. While x-axis shows the feature value calculated based on the expert’s annotation, y-axis determines the value based on the model’s prediction. The points on the line have zero errors.

**Figure 5:**
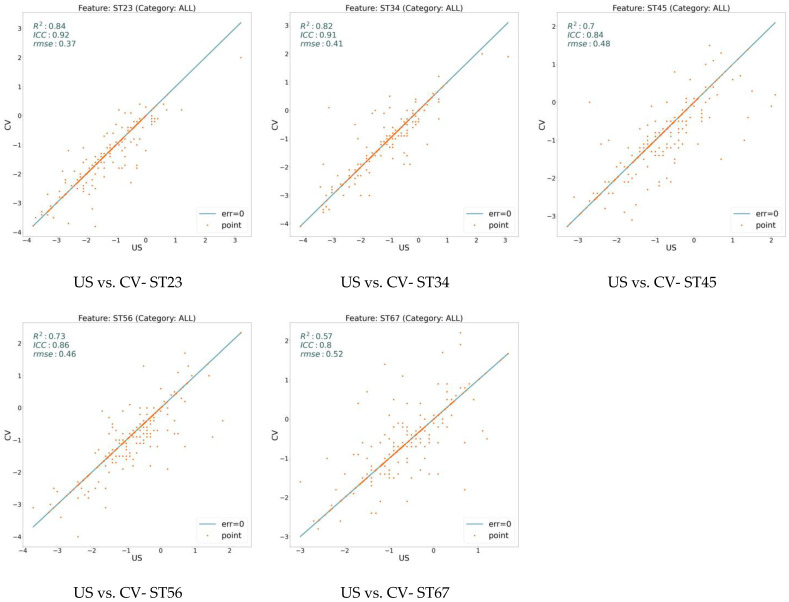
Error analysis between human expert (US) and the model (CV) over ST feature. While x-axis shows the feature value calculated based on the expert’s annotation, y-axis determines the value based on the model’s prediction. The points on the line have zero errors.

We extracted 18 variables for all our data sampling and analyzed them using three measures: RMSE, ICC, and R^2^. Figure 2 illustrates these values on the model (CV) with respect to the expert human (US) over three general features: ARA, C1H, and TR. On the other hand, Figure 3, Figure 4, and Figure 5 show the same measures for the segmental features, KA, RRA, and ST, respectively.

In Figure 2, Figure 3, Figure 4 and Figure 5, the line shows zero error, whereas the points with more distance from the line indicate more error. There is a direct relation between lower error and the measures ICC and R^2^, while the higher RMSE indicates more error. To distinguish the accuracy of the model on the different variables (features), we classify the features into three groups based on their R^2^ value. As shown in the figures, the R^2^ value for the features ARA, KA23, KA34, KA45, KA56, and TR is more than 0.90, so we classify them as the super-clean group of the features. However, R^2^ is between 0.75 and 0.90 for the features C1H, RR23, RR34, RR45, RR56, ST23, and ST34, which constitute the clean group of features. Finally, since the R^2^ score of the features KA67, RR67, ST45, ST56, and ST67 is between 0.5 and 0.75, they are considered semi-clean features.

The reported R^2^, ICC, and RMSE in Figure 2, Figure 3, Figure 4 and Figure 5 show that the error rate between the model (CV) and the expert human (US) annotator is not significant. While the R^2^ score is in the range of [0.57, 0.99], the ICC varies in the boundary of [0.80, 0.99]. These numbers are accompanied by the acceptable RMSE for all the proposed features. Based on the information shown in these figures, the error rate between the model and the expert human is negligible; therefore, the model is reliable. This reliability is also assessed by repeating the experiment of the annotation by an expert human twice.

**Figure 6:**
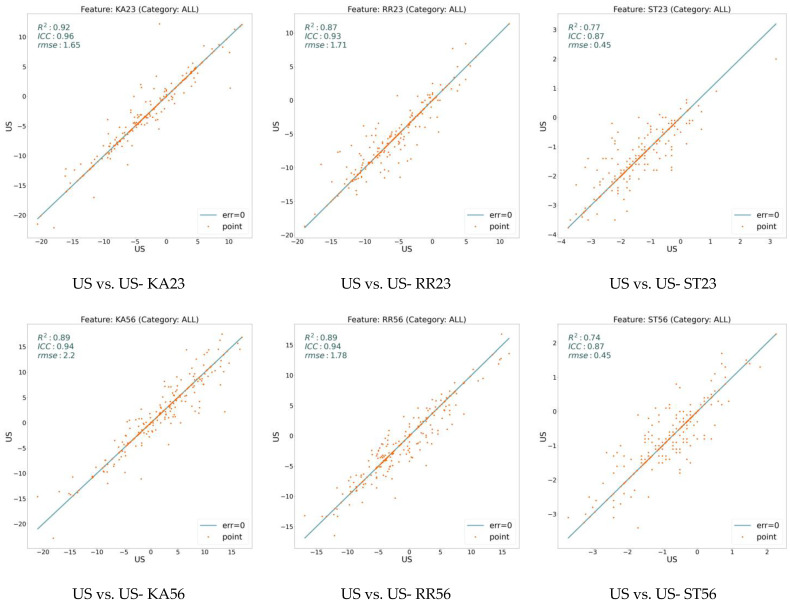
Error on two experiments by an expert human (US). While the x-axis shows the feature value calculated based on the expert’s first experiment, the y-axis determines the feature value based on the expert’s second experiment. The points on the line have zero errors. See the Methods section for description of the variables.

To assess the reliability of the model, every image was annotated two times by the same expert human at different times to study the intra-annotator error rate. Figure 6 reveals the error rate of the expert human on some of the randomly selected features. A comparison between the similar features of Figure 2, Figure 3, Figure 4 and Figure 5 and Figure 6 depicts that the error value between the model and the expert human is in the range of the error between the two experiments conducted by the expert human. For example, for the feature KA23, the R^2^ value for the model analysis is 0.93, while this measure for the expert analysis is 0.92. Studying the features in Figure 6 shows that among the six studied features, in the features KA23, KA56, and ST23, the agreement between the model and expert is even more than two experiments held by the expert human. In addition, Table 1 presents the mean and standard deviation of the error, as well as the root mean squared error, ICC, and R^2^ for both experiments between the model and human and human against human. This experimental result highlights the fact that the accuracy of the deep neural network model is comparable to the real expert human, and medical landmark detection issues can be trusted.

**Figure 7:**
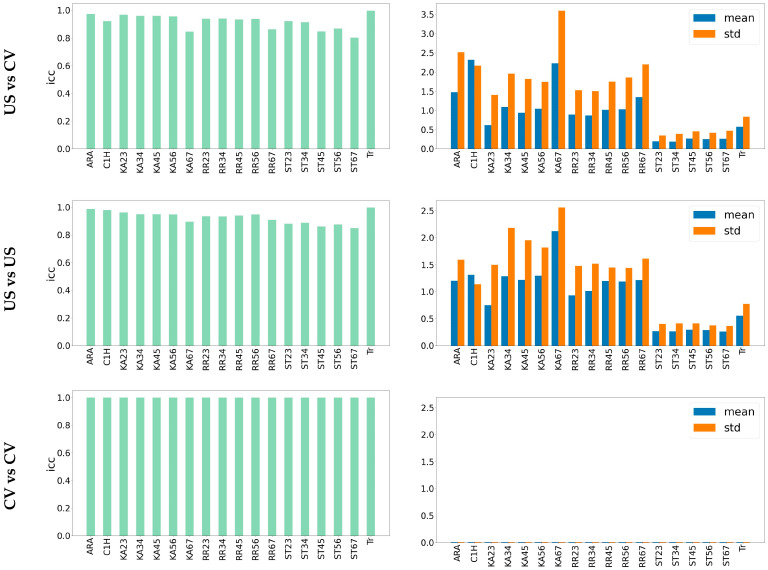
Diagram of the ICC, mean, and standard deviation of the error over different experiments. Comparing the diagrams related to US vs. CV and US vs. US shows that the error rate between these two experiments is very similar. Therefore, the model expectancy is the same as the human annotator. There is no difference between the model output in different experiments (CV vs. CV), so the error rate is zero, and ICC is maximum (ICC = 1).

Moreover, Figure 7 reveals similar discrepancies over different features in CV versus US and US versus US experiments. This similarity determines that the model’s variation is like the human annotator’s variation. This information indicates that the trained model is as reliable as the human expert annotator. It is notable that in the CV versus CV analysis, there is no error, and the ICC is maximum at 1.0. The reason is that the trained model, like all the other CNN models, is deterministic and always generates the same results for a specific input. To find some comparison between the baseline CNN model and the other models (CV vs. the other CVs), see [17].

## 4. Discussion

Deep learning models are widely used in automated medical image processing tasks, including image segmentation, tumor detection, and anatomical landmark point detection. In a recent study, we developed a Convolutional Neural Networks (CNNs) based model to automatically detect spine landmark points in the sagittal cervical spine on X-ray images [17]. Our developed model has been shown to be very accurate in predicting radiographic anatomical landmark vertebral points [17]. This prior investigation demonstrated that computer vision (CV) is superior to human measurement of spine displacements [17]. Our current investigation is a continuation of our previous study, where we provided comparisons with the current CNN model to other CNN models that exist in the literature, and the reader is referred to this background information [17]. 

In our current investigation, to further investigate the reliability and real-world validity of the model against a human annotator over time with repeated measures, we first collected reference data, annotated by an expert human annotator, and then compared the model’s predictions with these reference points. To further evaluate the annotation process, we also asked the annotator to repeat the annotations a second time two weeks later to further measure the annotator’s reliability. This allowed us to compare the trustworthiness of the model concerning the annotator’s reliability. Using 18 standard rotational and translational variable measurements for the sagittal cervical spine, our reliability results indicate good to excellent intra-class correlation coefficients (ICCs), small root mean squared errors (RMSE), and good to excellent to perfect R^2^ values, depending upon the variable assessed. Furthermore, our findings that the error rate between the two human user and computer vision experiments is very similar indicate that the computer vision model expectancy outcomes are the same as the human annotator. Furthermore, there is no difference between the CV model output under the two tested experiments, so the error rate is zero, the ICC is maximum, and the R^2^ value is perfect. Thus, both of our study’s hypotheses are validated in as much as the human and the CNN system have excellent intra-examiner reliability, and the CNN model has high construct validity compared to the experienced human.

Neck pain is a major contributor to the global burden of disease and is rated as the fourth greatest contributor to global disability [19]. Chronic neck pain is associated with reduced productivity and increased healthcare utilization and can lead to functional impairment and psychological distress, both of which can compromise overall quality of life [20]. There is a growing interest concerning the understanding of the biomechanics of the sagittal configuration of the cervical spine [21]. Importantly, in the past two decades, cervical sagittal alignment has gained more attention as an important clinical outcome in healthcare. It has been demonstrated that abnormal cervical sagittal alignment significantly influences human health and well-being, as it has been shown to be associated with pain [22], disability [23], overall functional performance [24], and quality of life [25]. Despite modern advances in technology related to imaging leading to improved diagnosis and treatment, billions of humans continue to suffer from daily spine and musculoskeletal pain and disability [11,26,27,28,29]. Physiotherapy, spinal manipulation, and exercise therapy have all been discussed as possible treatments for spine pain. However, these interventions typically do not have high-quality, long-term studies demonstrating successful improvements in HRQoL or patient-reported outcomes. Physical medicine and rehabilitation investigations have reported some positive pain outcomes but do not often report improvements in coronal and sagittal postural and spine balance parameters with the long-term stability of the successful intervention [30,31,32,33,34,35].

The diagnosis and treatment of spine pain and spinal trauma to determine the necessity for more invasive methods have been reported for many decades. Clinically, the use of X-ray for simple images of structure and tissues has been a consistently relied upon tool for spinal conditions causing pain. Reliable, repeatable, valid, and economical methods are necessary for the proper diagnosis of spine pain and associated conditions. Safe and efficacious treatment of spine conditions is a desirable clinical outcome for astute clinicians, physicians, surgeons, and therapists [36,37,38]. Cervical spine radiography provides physicians with a simple and repeatable method to determine sagittal and coronal balance, intersegmental spine misalignment, and differential diagnosis and frequently changes treatment options and approaches [36,37,38,39,40,41]. Specific spine rehabilitation protocols (based on radiographic measured variables) designed to lessen abnormal tissue loads via specific opposite posture exercises, spine extension traction, and spine manipulative therapy show potential for the treatment of spine pain and associated conditions using conservative and safe, repeatable, and efficacious methods [37,38,39,40]. These postural and structural rehabilitation investigation methods studied the sagittal spine configuration and developed average and ideal models for spine clinicians to use to make proper diagnosis and treatment recommendations based on the measurements [36,37,38,40,41].

The diagnosis and treatment of spine conditions have advanced with modern technology, and this technology has enabled advances in options for care. Digital radiography, computerized mensuration programs, and precision digitization tools are necessary to aid and reduce human error from both interventions and spine alignment diagnosis [14,15,16,17]. It has previously been shown that radiography mensuration techniques using analog tools such as pencils and protractors are repeatable, reliable, and valid with multiple investigations that show good inter- and intra-examinator agreement [42,43,44,45]. Radiographic measures of total cervical curvature (absolute rotation angle, ARA C2-C7, and Cobb angles) have previously been shown to have excellent examiner reliability [42,43]. For example, a recent meta-analysis identified that the Cobb method (inferior C2–inferior C7), the Cobb method (middle C1–inferior C7), and the absolute rotation angle (C2–C7) all have very high inter-rater reliability [42]. Similarly, relative rotation angles (RRA’s) for measurement of segmental cervical lordosis have been found to have excellent examiner reliability [44,45]. Finally, the measurement of anterior head translation (AHT) using the horizontal offset of C2 relative to a vertical line originating at the posterior inferior body of C7 has been found to have excellent reliability [44,45].

This current CNN model shows far superior accuracy to previously reported reliability investigations in as much as our results demonstrate a perfect R^2^ analysis, which is not reproducible with human evaluators even with great experience [42,43,44,45]. Likewise, when comparing the CNN model to itself in the repeated measures, the root mean squared errors (RMSE) were zero, and the ICCs were maximum (1.0), indicating perfect agreement with itself. To our knowledge, it has not been previously demonstrated that a cervical spine radiography CNN alignment tool can demonstrate such precision in the measurement of all 18 of the measured sagittal plane variables as performed herein. This is especially important when understanding that computer vision recognizes the lateral cervical radiograph every time and with exacting reliability and has demonstrated the ability to measure the structural abnormalities every time with an error of zero. There were no other programs in the literature that have computer vision networks that can recognize a lateral cervical radiograph every time and measure repeatably with such precision. Of note, the program appears to be learning much in the way that the human measurements improve over time. The clinical application of a tool such as this software should provide clinicians with much more certainty in their accurate diagnosis of spine abnormalities and likely improve the outcome of treatment due to less human error in the assessment and application.

In practical application, our original CNN model (and other CCN models) is a more accurate method of assessing anatomical and biomechanical positions of the cervical spine in the analysis of radiographic images as compared to a trained and experienced human user. These computerized analytical models have clear advantages over human capabilities. However, caution is advisable in this regard as the spine alignment data that these models derive and report are only one part of the healthcare physician’s basis in the formulation of conclusions and proper diagnoses for a given patient; the findings must be taken in the larger context of the full and comprehensive patient examination. It is the combination of medical knowledge and experience of the treating healthcare provider combined with image analysis using sophisticated CNN models that will result in a well-planned and executed treatment plan and procedures. Despite the recognition of sophisticated computerized examination methods as being more objective, with more precise measurements, the decisive variable in diagnosis and therapy application is the unique clinical presentation of the patient; thus, these CNN models and their enhanced measurements must be considered as an auxiliary tool for and not to replace the physician. Therefore, it is worth remembering that the human aspect of medicine has not lost and must not lose its importance.

### 4.1. Limitations

The limitations of this study are the fact that it is the first report of perfect accuracy with computer vision spine biomechanics mensuration, and repeated studies are necessary for firm conclusions. Further, larger studies are necessary to make absolute statements confirming the perfect accuracy of the program across various external datasets encompassing multiple spine conditions and surgical instrumented or fused segments. Larger studies incorporating radiography of other views of the spine and multiple spinal regions (full spine films) need to be performed as well, and investigations involving degeneration, congenital and morphological anomalies, as well as the consequences of single or multiple traumatic spine injuries should be performed [46]. Accordingly, larger studies are planned to involve more physicians’ images across multiple conditions using the software and CNN model. Studies involving patients with and without the use of PostureRay^®^ could further illuminate the necessity of precise alignment diagnosis before surgical and non-surgical interventions. However, it is noteworthy that the baseline model is trained over a set of images cropped with 5–10 percent boundaries around all the spines; therefore, cropping may affect the model performance. This is inevitable in machine learning tasks. The easiest solution to tackle this source of error is training a model to automatically crop a fixed area around the spine boundary.

### 4.2. Conclusions

A machine learning tool (which is part of the PostureRay^®^ software 2024) is simple, economical, valid, and repeatable as an instrument to aid in the measurement of the sagittal cervical spine alignment. Our investigation demonstrated the machine learning computer vision tool has a perfect R^2^ statistical analysis, a zero root mean square error, and an ICC of 1.0 (perfect reliability) when tested against itself with a repeated measure design. Additionally, the construct validity of the CNN software 2024 compared to an expert annotator was in the excellent range. This easy-to-use tool is far superior with regards to reliability when compared to analysis by a human clinician, even with many years of radiographic mensuration experience both manually and digitally and the tool appears to be perfect relative to itself every time, unlike the human. To our knowledge, this excellent reliability and validity has not been previously reported in the machine learning literature. Additional research is warranted to determine full spine condition implications for this technology.

## Figures and Tables

**Table 1:** Details of different measures on various measurements reported. Mean: mean; Std: standard deviation of the error; RMSE: root mean squared error; ICC: intraclass correlation coefficients. Features (variables) include ARA: absolute rotation angle; C1H: atlas plane line to horizontal; KA: Cobb angle endplate lines from C2/C3 inclusive to C6/C7; RRA: posterior body tangent lines from C2/C3 inclusive to C6/C7; ST: intersegmental sagittal translation distance from C2/C3 inclusive to C6/C7; TR: global sagittal translation distance of C2 relative to C7.

	Computer Vision (CV) vs. Expert Human (US)					Expert Human (US) vs. Expert Human (US)				
	Mean	Std	RMSE	ICC	R^2^	Mean	Std	RMSE	ICC	R^2^
ARA (°)	1.13	0.014	2.89	0.97	0.94	0.89	0.009	1.97	0.98	0.97
C1H (°)	1.48	0.018	2.48	0.92	0.86	0.93	0.012	1.66	0.97	0.95
KA23 (°)	0.55	0.015	1.48	0.96	0.93	0.59	0.017	1.65	0.96	0.92
KA34 (°)	0.93	0.017	2.14	0.95	0.91	1.05	0.019	2.46	0.94	0.89
KA45 (°)	0.80	0.017	1.99	0.95	0.91	1.00	0.019	2.25	0.94	0.89
KA56 (°)	0.85	0.018	1.98	0.95	0.91	0.98	0.020	2.20	0.94	0.89
KA67 (°)	1.74	0.037	4.18	0.84	0.64	1.59	0.028	3.27	0.89	0.78
RRA23 (°)	0.66	0.022	1.76	0.93	0.86	0.75	0.022	1.71	0.93	0.87
RRA34 (°)	0.72	0.021	1.69	0.94	0.88	0.83	0.021	1.76	0.93	0.86
RRA45 (°)	0.80	0.023	2.00	0.93	0.86	0.95	0.020	1.79	0.93	0.88
RRA56 (°)	0.79	0.022	2.11	0.93	0.86	0.94	0.019	1.78	0.94	0.89
RRA67 (°)	1.09	0.033	2.53	0.86	0.70	0.97	0.025	1.93	0.90	0.82
ST23 (mm)	0.16	0.023	0.37	0.92	0.84	0.23	0.028	0.45	0.87	0.77
ST34 (mm)	0.16	0.025	0.41	0.91	0.82	0.22	0.028	0.46	0.88	0.77
ST45 (mm)	0.24	0.031	0.48	0.84	0.70	0.24	0.031	0.49	0.86	0.72
ST56 (mm)	0.21	0.031	0.46	0.86	0.73	0.22	0.030	0.45	0.87	0.74
ST67 (mm)	0.23	0.039	0.52	0.80	0.57	0.22	0.032	0.42	0.84	0.70
TR (mm)	0.45	0.003	0.96	0.99	0.99	0.44	0.003	0.87	0.99	0.99

## Data Availability

The datasets analyzed in the current study are available from the corresponding authors upon reasonable request.
